# MicroRNA modulate alveolar epithelial response to cyclic stretch

**DOI:** 10.1186/1471-2164-13-154

**Published:** 2012-04-26

**Authors:** Nadir Yehya, Adi Yerrapureddy, John Tobias, Susan S Margulies

**Affiliations:** 1 Department of Bioengineering, University of Pennsylvania, 240 Skirkanich Hall, 210 South 33rd Street, Philadelphia, PA, 19104-6321, USA; 2 Division of Anesthesiology and Critical Care, Children’s Hospital of Philadelphia, Philadelphia, PA, 19104, USA; 3 Bioinformatics Core, University of Pennsylvania, Philadelphia, PA, 19104, USA

## Abstract

**Background:**

MicroRNAs (miRNAs) are post-transcriptional regulators of gene expression implicated in multiple cellular processes. Cyclic stretch of alveoli is characteristic of mechanical ventilation, and is postulated to be partly responsible for the lung injury and inflammation in ventilator-induced lung injury. We propose that miRNAs may regulate some of the stretch response, and therefore hypothesized that miRNAs would be differentially expressed between cyclically stretched and unstretched rat alveolar epithelial cells (RAECs).

**Results:**

RAECs were isolated and cultured to express type I epithelial characteristics. They were then equibiaxially stretched to 25% change in surface area at 15 cycles/minute for 1 hour or 6 hours, or served as unstretched controls, and miRNAs were extracted. Expression profiling of the miRNAs with at least 1.5-fold change over controls revealed 42 miRNAs were regulated (34 up and 8 down) with stretch. We validated 6 of the miRNAs using real-time PCR. Using a parallel mRNA array under identical conditions and publicly available databases, target genes for these 42 differentially regulated miRNAs were identified. Many of these genes had significant up- or down-regulation under the same stretch conditions. There were 362 down-regulated genes associated with up-regulated miRNAs, and 101 up-regulated genes associated with down-regulated miRNAs. Specific inhibition of two selected miRNAs demonstrated a reduction of the increased epithelial permeability seen with cyclic stretch.

**Conclusions:**

We conclude that miRNA expression is differentially expressed between cyclically stretched and unstretched alveolar epithelial cells, and may offer opportunities for therapeutic intervention to ameliorate stretch-associated alveolar epithelial cell dysfunction.

## Background

Mechanical ventilation of patients with respiratory failure is known to increase alveolar epithelial permeability [1-3] and initiate an inflammatory response [4,5], which contributes to the elevated morbidity and mortality seen in these patients [6-8]. Lower tidal volume ventilation may improve survival as compared with higher tidal volume ventilation [9], suggesting that smaller cyclic stretches with less alveolar cell deformation may mitigate some of the damage of ventilator-induced lung injury (VILI).

The mechanisms behind the increased permeability remain unclear. Previous studies have shown decreased protein content at tight junctions of cyclically stretched epithelia [10], disorganization of actin monofilaments [10,11], and elevated intracellular calcium concentrations [12]. Genomic analysis of tissue homogenates from whole animals [13-16] and intact mouse lungs [17] exposed to large tidal volume ventilation consolidate the responses of multiple cell types, including endothelial, epithelial, and inflammatory leukocytes. Our group has recently reported on the genomic response of type I epithelia, which comprise >95% of the surface area of the alveolus. Cultured primary alveolar epithelial cells with type I characteristics were exposed to low and moderate stretch magnitudes (change in surface area, ΔSA, of 12% or 25%), and varying durations of cyclic stretch (1 hour or 6 hours) relative to unstretched cells [18]. Both magnitude- and duration-dependent gene expression changes were evident, implicating several genes previously unknown to be affected by either *in vitro* stretch or VILI.

MicroRNAs (miRNAs) are a class of small noncoding RNAs implicated in multiple physiologic processes via negative post-transcriptional regulation of messenger RNAs (mRNAs). The transcription of miRNAs is under similar control as that of mRNAs, and their expression can be similarly profiled [19]. MiRNAs have been implicated in the proliferation and differentiation of myocytes in response to cyclic stretch [20], suggesting a possible role for stretch in other cell types. MiRNAs are involved in the hypoxic response of many cell types [21-24] and the post-infarct myocardium [25-27], and also in the fibrotic response after ischemia/reperfusion [28,29], consistent with their role as modifiers of tissue injury and healing. Because miRNAs modulate responses, they may offer potential as therapeutic interventions. Several features of miRNAs make them attractive as therapies: miRNAs can be efficiently stabilized or inhibited [30,31]; and, some miRNAs regulate multiple mRNAs, and can therefore potentially modify entire gene networks [30].

Previously, our group developed an *in vitro* monolayer with alveolar type I characteristics that mimic lung inflation when subjected to equibiaxial stretch [7,8,10,32]. Using this model, we identified the genome-wide miRNA expression profile of these cells when subjected to different durations (1 hour or 6 hours) of cyclic stretch at a magnitude of 25% ΔSA. Using publicly available predictive databases [33], we identified likely mRNA targets of these miRNAs, and further refined our list by concentrating on mRNAs previously demonstrated to be differentially expressed in this same cyclically-stretched monolayer model [18]. Finally, we demonstrated that specific inhibition of two select miRNAs up-regulated with stretch partially ameliorates the phenotype of increased epithelial permeability seen with cyclic stretch, suggesting a functional role for these miRNAs in regulating the cell response to stretch.

## Methods

### Primary Rat Alveolar Epithelial Cell (RAEC) isolation

In a protocol approved by the Animal Care and Use Committee of the University of Pennsylvania, alveolar type II cells were isolated from male Sprague–Dawley rats based on a method reported by Dobbs et al. [34], with the slight modification reported earlier [35]. Type II cells were seeded onto fibronectin-coated (10 μg/cm^2^) flexible silastic membranes (Specialty Manufacturing, Saginaw, MI), mounted in custom-designed wells at a density of 10^6^ cells/cm^2^. The cells were cultured for 5 days with MEM supplemented with 10% fetal bovine serum, until they attained alveolar type I cell characteristics [32]. Then these RAEC were serum-deprived with 20 mM Hepes supplemented with DMEM (CO_2_-free buffering system) for 2 hours, subjected to biaxial cyclic stretch (25% ∆SA) at 37°C for one of two durations (1 hour or 6 hours) at a frequency of 0.25 Hz (15 cycles/min). The two stretch groups were designated 25*1 and 25*6. All the samples were compared to unstretched control wells.

### Total RNA isolation

Total RNA was extracted from the cells (Qiagen miRN easy mini kit cat# 217004, Qiagen Inc, Valencia, CA) according to the manufacturer’s instructions. RNA samples were obtained from every experimental group. The quantity and quality of the RNA samples were measured (Agilent Bioanalyzer and Nanodrop spectrophotometer). Samples with low RNA integrity number were discarded. The final group used in the microarray analysis included 12 samples, with N = 4 animals/group. The miRNA microarray protocols were conducted as described in the Exiqon LNA microRNA Amplification protocol at University of Pennsylvania Microarray Core Facility, and the raw miRNA expression data were evaluated.

### MiRNA microarray data analysis

The miRNA expression data were imported into Partek Genomics Suite (v6.4, Partek Inc., St. Louis, MO). The probes were filtered for chip-to-chip differences with Loess non-linear normalization [36], and only those probes with a significant detection (p < 0.05) in at least 3 of the 12 samples were retained, leaving 1279 for subsequent analysis. The intensity values of the remaining probes were transformed (log_2_). Pairwise contrasts between the control and each of the two stretch groups (25*1 and 25*6) were also determined in a post-hoc analysis. No significant array or animal effect was observed in the data. The complete miRNA expression microarray data set is available via Gene Expression Omnibus accession number GSE36256.

To attribute significance to any of the p-values calculated in the ANOVA and pairwise contrasts, we used a 10% False Discovery Rate (FDR, Benjamini-Hochberg, step-up) cutoff for analysis of the data [37]. Based on this criterion, there were a total of 52 miRNAs with at least one significant p-value in the two pairwise comparisons. This miRNA set was further filtered to investigate only those miRNAs with expressions that were altered at least 1.5-fold up or down in at least one of the comparisons with controls, leaving 42 miRNAs for subsequent analyses.

### Target prediction

To identify the possible gene targets that are regulated by these 42 miRNAs, we used two publicly available target prediction databases: TargetScan 5.1 [38] and MicroCosm Targets 5.0. We limited our analysis to those gene targets predicted by both databases. TargetScan assigns a context score and context percentile to each predicted gene target for a particular miRNA, and we used a TargetScan cutoff of ≥50^th^ context percentile. We defined any of our 42 miRNAs that regulated more than 5 genes as “promiscuous.” Because the predicted target gene population for the 42 miRNAs of interest was very large, we focused our analysis on only those gene targets that were regulated significantly (FDR ≤ 0.1) in our earlier rat alveolar epithelial cell stretch study [18], where we reported 3681 genes that are differentially expressed after 6 hours of cyclic stretch at 25% ΔSA, compared with unstretched controls (FDR ≤ 0.1). Limiting our analysis to miRNA gene targets that were among those 3681 genes, and which were anti-correlated with the miRNAs in this study (i.e., up-regulated miRNAs were targeted to down-regulated mRNAs predicted by both MicroCosm and TargetScan ≥50^th^ context percentile), we distilled our set down to 463 gene targets of greatest interest. These 463 genes were subjected to enrichment analysis using the Database for Annotation, Visualization and Integrated Discovery (DAVID) v6.7 functional annotation tool [39]. The 463 genes of interest were grouped according to their Kyoto Encyclopedia of Genes and Genomes (KEGG) pathways, and were checked for overrepresentation relative to a background gene list comprising the 3681 genes differentially expressed with stretch. An Expression Analysis Systematic Explorer (EASE) score from a modified Fisher exact test was calculated for each KEGG grouping, with an EASE score of <0.05 considered significant.

### Real-time PCR assay

To validate our miRNA microarray findings, we performed real-time PCR on 6 miRNAs of interest. Using the methods described above, miRNAs were obtained from rat alveolar epithelial cell isolations maintained in culture for 5 days, and were either stretched at 25% ∆SA for 6 hours (25*6) or designated as unstretched controls. Five of these six miRNAs (miR-423-5p, miR-466d-5p, miR-378, miR-32* and miR-15b) were validated via first-strand cDNA synthesis in 20-μl reactions with sequence-specific Taqman miRNA primers (Taqman MicroRNA reverse transcription kit) used according to the manufacturer’s protocol. For the sixth miRNA (miR-466f-3p), we used an NCode SYBR Green miRNA qRT-PCR Kit (Invitrogen MIRQ-100). Real-time PCR was performed (Taqman universal master mix or Applied Biosystems power SYBR green PCR master mix). Rat 4.5 s RNA was evaluated and used as an endogenous control for the miRNAs.

### MiRNA inhibition and permeability assay

To validate the functional significance of identified miRNAs, we chose two representative miRNAs up-regulated with stretch (miR-466d-5p and miR-466f-3p). For each of these two miRNAs, we transfected RAECs with custom-designed commercially available inhibitors prior to stretch, and assayed the monolayer permeability. Specifically, using the above-described methods, we isolated RAECs and kept them in culture for 3 days in antibiotic-free MEM supplemented with 10% fetal bovine serum. On the fourth day of culture, we transfected custom-designed, specific inhibitors of miR-466d-5p, miR-466f-3p, or a scrambled negative control (Exiquon). These inhibitors use a locked nucleic acid (LNA) technology, and were conjugated to a Texas Red dye for visual confirmation of transfection efficiency. RAECs were transfected with miRNA inhibitors or the negative control using Lipofectamine 2000 (Invitrogen) reagent diluted in Opti-MEM for a final miRNA inhibitor concentration of 80 nM, per manufacturer’s protocol. Optimum transfection concentration was determined by titration of different concentrations of inhibitors (50 to 100 nM) and an evaluation of monolayer fluorescence after 24 hours, using >90% cellular fluorescence as a threshold for efficient transfection. On the fifth day of the culture, RAECs were serum-deprived with 20 mM Hepes supplemented with DMEM for 2 hours, subjected to biaxial cyclic stretch (25% ∆SA) at 37°C for 6 hours at a frequency of 0.25 Hz (15 cycles/min), or served as unstretched controls, with N = 4 animals/group.

To assay for RAEC permeability, we used a small uncharged molecule ouabain, with high affinity for the basolateral Na^+^/K^+^-ATPase, conjugated to a nonpolar fluorophore BODIPY, as a tracer. Our group has validated this method before [8], and demonstrated that BODIPY-ouabain binding represents loss of tight junction integrity and increased paracellular permeability, and that increased fluorescent signal is not a result of cell death or intracellular uptake or transport. After 5 of the 6 hours of equibiaxial stretch (or no stretch for controls), BODIPY-ouabain (Invitrogen) diluted in dimethyl sulfoxide was added to all cells at a final concentration of 2 μM, and incubated for the final 1 hour of stretch. At the end of the hour, all cells were washed with DMEM to remove excess BODIPY-ouabain from the apical surface, and were visualized under a fluorescent microscope using a green emission filter. Presence of fluorescence was evidence of high affinity ouabain binding to the basolateral surface, and was used as a measure of paracellular permeability. Fluorescence was measured on 4 separate fields per well, and 3 wells were measured per treatment group per animal. All measurements were normalized to values from unstretched, untransfected cells.

## Results

In a pairwise manner, the two stretch duration groups (25*1 and 25*6) were compared to unstretched controls. At FDR ≤ 0.1 and a minimum of 1.5-fold increase or decrease, 1 miRNA was differentially expressed at 1 hour of stretch, and 42 miRNAs were differentially expressed at 6 hours of stretch. The single miRNA significantly up-regulated at 1 hour (miR-423-5p) remained up-regulated at 6 hours; another 33 miRNAs were significantly up-regulated and another 8 miRNAs were significantly down-regulated at 6 hours.

The 5 miRNAs with the greatest fold-change in up- or down-regulation at 6 hours of stretch are presented in Table 1. The highest fold-change was a 2.9-fold increase in miR-32*. The miRNA* designates the complementary “passenger” transcript on the hairpin arm of the precursor miRNA opposite the mature miRNA, and after cleavage these usually exist at a lower frequency than the mature miRNA [19]. Their biological relevance remains unclear. In our array, miR-32* is significantly up-regulated with stretch, while miR-32 is not significantly differentially expressed, suggesting the possibility that miR-32* may be the primary transcript or may have important functional significance.

**Table 1:** Five largest up- and down-regulated miRNAs

miRNA probe	Fold change	Probable genes^a^ predicted by both algorithms
miR-32*^b^	2.9	—
miR-466c-5p	2.8	22
miR-466d-5p	2.8	30
miR-466c	2.6	4
miR-466b	2.6	7
miR-466f-3p^c^	2.4	38
miR-375	−1.7	13
miR-378	−1.6	18
miR-347	−1.6	23
miR-15b	−1.6	21
miR-154	−1.5	16

^a^ Probable gene targets are candidates from a parallel mRNA expression array differentially expressed with cyclic stretch of 25% ΔSA at 6 hours, and predicted by both MicroCosm and TargetScan algorithms; see text for details.

^b^ The majority of miRNAs with the “*” designation are considered passenger transcripts of a precursor miRNA, and do not have calculated TargetScan targets.

^c^ MiR-466f-3p is the 7^th^ highest up-regulated miRNA, and was chosen for subsequent validation studies; it is included here for reference.

**Figure 1:**
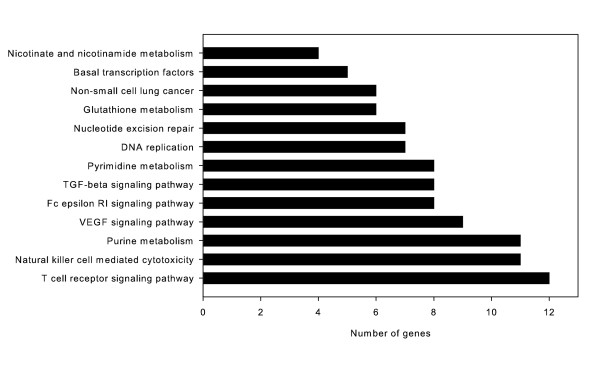
**DAVID functional annotation.** The 362 predicted gene targets of up-regulated miRNAs, and the 101 predicted gene targets of down-regulated miRNAs are grouped according to KEGG pathways with an enrichment EASE score < 0.05 relative to background gene list comprising all 3681 genes significantly differentially expressed with cyclic stretch.

The functional significance of differential miRNA expression in cyclic stretch was determined using two publicly available databases. TargetScan 5.1, which predicts biological targets of mammalian miRNAs using seed-site complementarity and 3′UTR site conservation, ranks putative targets with a context score and percentile. Increasing context percentiles further restricts the number of potential target genes. TargetScan ignores most miRNA* sequences. MicroCosm Targets (formerly miRBase Targets) uses the miRanda algorithm to identify potential targets, and requires strict complementarity at the seed region as well as 3′UTR site conservation. Potential target genes must be predicted by both algorithms. In order to further refine our list of potential miRNA targets, we restricted our gene list to mRNA demonstrated to be differentially expressed (3681 genes at FDR ≤ 0.1) at 25% ΔSA at 6 hours in a previous mRNA expression analysis under identical conditions [18], and anti-correlated with the direction of miRNA expression. Mechanistically, miRNAs inhibit mRNA translation, but also are often associated with a down-regulation of their mRNA targets [40,41]; therefore, we felt that focusing on inversely related miRNA:mRNA pairings was justified. Using these criteria provided a list of 362 high probability down-regulated gene targets of up-regulated miRNAs, and 101 up-regulated targets of down-regulated miRNAs. Functional annotation of these 463 genes using the DAVID tool showed multiple pathways from KEGG were significantly overrepresented (Enrichment Analysis Systematic Explorer, EASE, score < 0.05) relative to a background of all 3681 genes significantly expressed with cyclic stretch (Figure 1, Table 2). Several groups are attractive candidates for pathways involved with cyclic stretch, such as the vascular endothelial growth factor (VEGF) and transforming growth factor-β (TGF-β) signaling pathways, and glutathione metabolism. Interestingly, all overrepresented pathways are comprised of genes targeted by up-regulated miRNAs.

**Table 2:** DAVID functional annotation

KEGG^a^ term	EASE^b^ score	Gene symbols
T cell receptor signaling pathway	0.002	ITK, PTPRC, PLCG1, CD8A, MAPK14, PIK3CD, SOS2, MAPK3, LCK, NFAT5, NFATC4, LCP2
Natural killer cell mediated cytotoxicity	0.003	CD48, PLCG1, PIK3CD, SOS2, MAPK3, LCK, NFAT5, NFATC4, SHC1, LCP2, NCR3
Purine metabolism	0.05	POLD3, POLE4, GDA, PDE6D, POLR3H, POLE2, ENPP1, NT5M, PDE8A, POLR2C, NME7
VEGF signaling pathway	0.004	SH2D2A, PLCG1, CASP9, MAPK14, PIK3CD, MAPK3, NFAT5, PLA2G2A, NFATC4
Fc epsilon RI signaling pathway	0.02	PLCG1, MAP2K3, MAPK14, PIK3CD, SOS2, MAPK3, PLA2G2A, LCP2
TGF-β signaling pathway	0.03	BMP4, INHBB, BMP2, RBL2, E2F5, ID2, TGFBR1, MAPK3
Pyrimidine metabolism	0.04	POLD3, POLE4, POLR3H, POLE2, NT5M, POLR2C, NME7, TK2
DNA replication	0.001	POLD3, POLE4, POLE2, PCNA, RNASEH1, RNASEH2A, MCM5
Nucleotide excision repair	0.004	POLD3, XPA, POLE4, POLE2, PCNA, CETN2, GTF2H2
Glutathione metabolism	0.03	GPX2, GSR, GPX3, GSTT2, ANPEP, MGST1
Non-small cell lung cancer	0.04	PDPK1, PLCG1, CASP9, PIK3CD, SOS2, MAPK3
Basal transcription factors	0.03	TAF2, GTF2E1, GTF2E2, TAF13, GTF2H2
Nicotinate and nicotinamide metabolism	0.05	NNT, ENPP1, NT5M, BST1

^a^ Kyoto Encyclopedia of Genes and Genomes.

^b^ Expression Analysis Systematic Explorer, a modified Fisher exact test.

**Figure 2:**
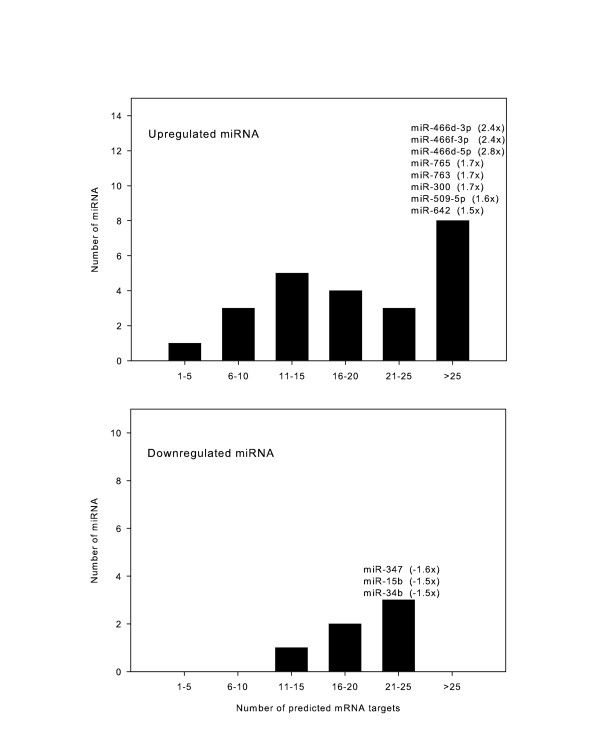
**“Promiscuous” miRNAs with multiple predicted mRNA targets.** The 34 up-regulated (top) and 8 down-regulated (bottom) miRNAs were matched with predicted high probability mRNA targets from a parallel mRNA expression array using TargetScan and MicroCosm. Most miRNAs had multiple predicted gene targets. Of the 34 up-regulated miRNAs, 8 had >25 predicted targets each; of the 8 down-regulated miRNAs, only 3 had >20 predicted targets.

**Figure 3:**
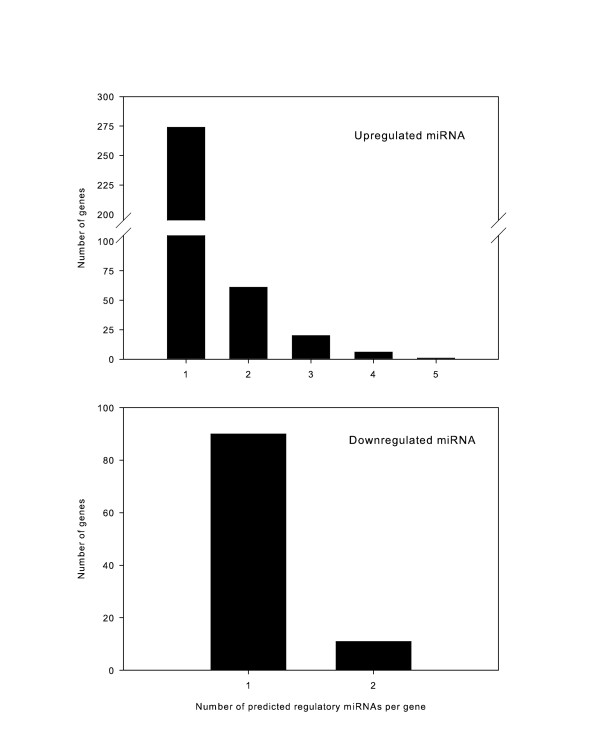
**“Redundant” genes with multiple miRNA target sites within their 3’ UTR.** The 362 high probability genes associated with the 34 up-regulated miRNAs (top) and the 101 genes associated with the 8 down-regulated miRNAs (bottom) are graphed according to the number of predicted miRNA:mRNA matches. While most genes are predicted to have been targeted by only a single miRNA, the genes associated with the up-regulated miRNAs show more redundancy, with 27 genes demonstrating ≥ 3 predicted miRNA:mRNA matches.

Several miRNAs targeted multiple mRNAs associated with cyclic stretch, which we defined as “promiscuous” miRNAs (Figure 2). Of special interest are three of the listed up-regulated miRNAs (miR-466d-3p, miR-466d-5p, and miR-466f-3p), which are highly expressed (> 2-fold) as well as promiscuous, with over 25 predicted mRNA targets each. Conversely, certain genes were predicted to be potential targets of several miRNAs, suggesting that the effect of miRNAs on the expression levels of these mRNAs may be “redundant” (Figure 3).

**Figure 4:**
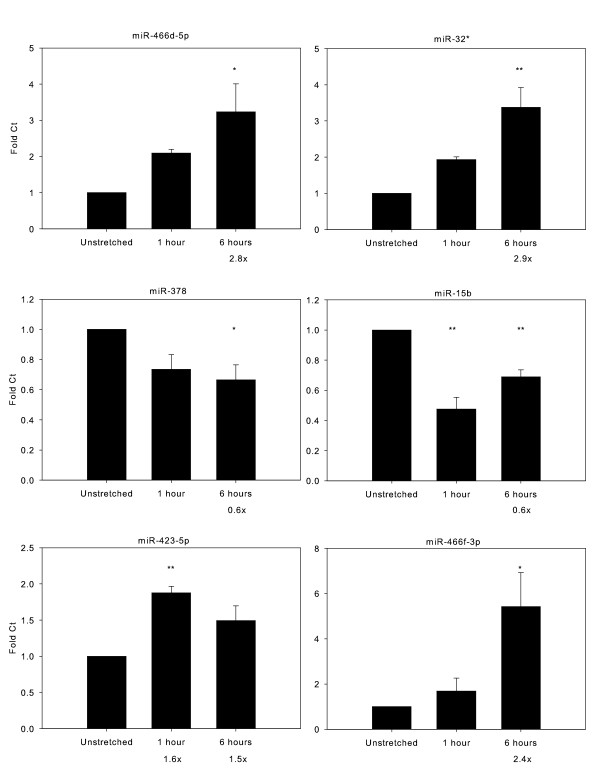
**Quantitative real-time PCR validation of miRNAs.** To validate differential expression of the miRNAs, 4 of the miRNAs with the largest fold-changes (2 up- and 2 down-regulated; see Table 1), were validated using quantitative real-time PCR with either Taqman or SYBR Green. In addition, we performed qRT-PCR on miR-423-5p, which was the only miRNA differentially expressed at both 1 and 6 hours, and miR-466f-3p, which was chosen for subsequent investigations. If significant (FDR of 10%), miRNA fold-change on the array is given below the corresponding stretch duration. PCR results are qualitatively consistent in direction and magnitude with the array. N = 4-6/group. ANOVA and post-hoc Tukey results: *p < 0.05; **p < 0.01, relative to unstretched. Expression was normalized to 4.5 s RNA.

Four of the miRNAs with large fold-changes (2 up- and 2 down-regulated) were validated using quantitative real-time PCR. In addition, we validated miR-423-5p, which was the only miRNA up-regulated ≥ 1.5-fold at both 1 and 6 hours of cyclic stretch. We also validated miR-446f-3p, which was up-regulated 2.4-fold after 6 hours of stretch and was the most promiscuous, with multiple predicted targets in known pathways implicated in VILI and alveolar stretch [42]. At both 1 hour and 6 hours of stretch at 25% ΔSA, the PCR results were qualitatively consistent in magnitude and direction with the expression array (Figure 4).

**Figure 5:**
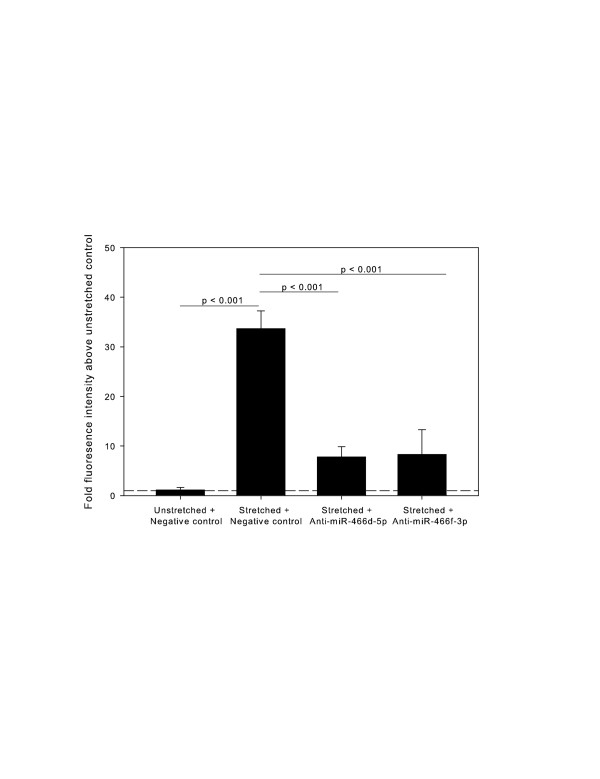
**Effect of miRNA inhibition on permeability of RAEC monolayer.** Cells were transfected with specific inhibitors to miR-466d-5p and miR-466f-3p, or a scrambled negative control, and subjected to stretch or served as unstretched controls. Permeability is expressed as fluorescence of BODIPY-ouabain tracer above baseline. Results are reported as mean ± SE and are normalized to the reference bar (set at 1), which represents the fluorescence of unstretched, untransfected cells. There is a large increase in permeability with cyclic stretch, which is partly ameliorated by specific miRNA inhibition. N = 4/group. P values are results of ANOVA and post-hoc Tukey.

In order to test the functional significance of our miRNA expression array, 2 miRNAs were chosen to be knocked down using commercially available specific inhibitors. We chose miR-466d-5p and miR-466f-3p because of their relative high expression (> 2-fold) and their promiscuity (> 25 predicted target mRNAs each). Specific inhibition of these 2 miRNAs was associated with a reduction in the cyclic-stretch–induced increase in permeability back to baseline unstretched levels, as measured by a reduction in the amount of basolateral binding of the BODIPY-ouabain tracer (Figure 5).

## Discussion

Primary alveolar epithelia with type I characteristics display a duration-dependent miRNA expression profile in response to cyclic stretch, with a single significantly up-regulated miRNA with  ≥ 1.5-fold change at 1 hour of stretch, but 34 up- and 8 down-regulated at 6 hours, compared with unstretched. These results are consistent with our previous demonstration of increased mRNA expression differences after 6 hours of stretch relative to 1 hour [18], suggesting an enhanced genomic response after sustained cyclic stretch involving both mRNA and miRNA expression. Six of the most informative miRNAs were validated by real-time reverse transcription PCR.

The highest fold-change in our array occurred in miR-32*, designated a passenger transcript, and therefore not a candidate for analysis with TargetScan 5.1. Passenger transcripts are typically degraded, and generally exist at significantly lower concentrations than their corresponding mature miRNAs [19]. However, several recent reports have suggested a role for miRNA* transcripts at critical junctures in several biological processes, ranging from miRNA and 3′UTR evolution [43] to apoptosis in malignant cells [44,45]. Therefore, despite their lower concentrations, miRNA* may perform important biological functions. In our array, miR-32* is 2.9-fold up-regulated with stretch, while miR-32 is not significantly differentially expressed, suggesting the possibility that miR-32* may, in fact, be the primary transcript. Direct scanning of the genome for matching 3′UTRs may reveal interesting candidate gene targets for miR-32*.

The predicted gene list was limited to mRNA predicted by both MicroCosm and TargetScan with a context percentile ≥ 50%, and further refined to those genes with significant differential expression with stretch in a concurrent mRNA microarray [18] and anti-correlated with the direction of miRNA expression. Using this approach, we identified genes predicted to be high-likelihood targets of miRNAs: 362 for up-regulated miRNAs, and 101 for down-regulated miRNAs. Functional annotation of likely gene targets for these miRNAs suggests a role for several signaling pathways to be modulated by miRNAs in response to stress and deformation. Restricting our analysis to genes differentially expressed in a parallel mRNA expression may allow for better prediction of meaningful targets of significant miRNAs. We further classified these miRNA:mRNA interactions for their promiscuity (multiple predicted gene targets) and redundancy (multiple miRNAs with a shared target gene). While some human miRNAs are known to have multiple predicted targets, most are predicted to control only a few genes [46]. This classification of our miRNAs allows us to generate hypotheses focusing on highly promiscuous miRNAs with multiple gene targets.

Functional annotation of gene targets showed overrepresentation of multiple pathways potentially significant in cyclic stretch. Previous work [47] has demonstrated that low-magnitude cyclic and sustained large-magnitude stretch can rapidly induce expression of extracellular signal-related kinase (ERK1/2-MAPK). The up-regulated miRNAs in our array are associated with an overrepresentation of gene targets in the VEGF signaling pathway upstream of MAPK, suggesting a possible role for miRNAs in regulating this response to stretch. Other overrepresented pathways, such as glutathione metabolism, have also been implicated in the alveolar response to stretch [48], suggesting a role for miRNAs in regulating oxidant release with stretch. Interestingly, the enriched pathways were populated by gene targets associated with only the miRNAs up-regulated with stretch, suggesting that the up-regulated miRNAs may contribute more to pathway modulation than down-regulated miRNAs. This is supported by the higher fold-changes for the up-regulated miRNAs.

Finally, we attempted to link the expression of the miRNAs during cyclic stretch with a phenotype known to be induced by stretch. Subjecting RAECs to cyclic stretch causes a loss of tight junction integrity and leads to increased paracellular permeability. Specific inhibition of miR-466d-5p and miR-466f-3p resulted in a preservation of barrier permeability close to baseline unstretched conditions, suggesting a role for these miRNAs in regulating the RAEC response to cyclic stretch. Future investigations will address the mechanism behind this reduced permeability. Of note, cyclic stretch leading to an increase in reactive oxygen species [48] and a reorganization of the cytoskeleton [49] with loss of tight junction integrity [10,50,51] has been separately implicated in leading to increased epithelial permeability. Our study allows the investigation of specific miRNA:mRNA interactions based on anti-correlated expression, and allows us to focus on known oxidative stress and cytoskeletal pathway mRNAs as attractive potential intermediaries between miRNAs and phenotypes.

Our study has several strengths. It represents the first systematic investigation of the role of miRNAs in the medically relevant process of cyclic stretch in alveolar epithelial cells, has demonstrated a link between miRNA expression and a phenotype (increased monolayer permeability), and has identified pathways for further investigation. By restricting our analyses to genes known to be differentially expressed with cyclic stretch from a parallel mRNA microarray experiment, we believe we have increased the likelihood of detecting relevant miRNA:mRNA interactions. Also, the organization of miRNAs and their targets as “promiscuous” or “redundant” allowed for more flexible investigation of the data, and allowed us to focus on miRNAs with potentially higher impact targets, or those with multiple targets. One of the theoretical advantages of knowing miRNA differential expression is the ability to potentially investigate the regulation of multiple genes simultaneously. The fact that the inhibition of two of the identified up-regulated miRNAs resulted in a dramatic amelioration of the increased permeability seen with cyclic stretch suggests regulation of multiple genes, or gene pathways, rather than control of a single locus.

Our study is limited by the fact that many of our predicted miRNA:mRNA interactions remain *in silico* only, and require a separate proof of regulation on an individual basis for any given interaction. However, this is somewhat mitigated by the use of two separate prediction algorithms to identify potential targets, as well as focusing on genes known to be expressed in stretched alveolar cells. We are also somewhat limited by the relatively low expression levels of the miRNAs in stretched cells relative to unstretched (<3-fold), making it difficult to link robust miRNA expression or inhibition with a phenotype. We are encouraged, however, by the clear effect on permeability seen after the specific inhibition of miR-466d-5p and miR-466f-3p. Overall, our data set provides a starting point for investigating high-value miRNAs predicted to regulate multiple target genes of interest.

## Conclusions

In summary, we have investigated and described the genome-wide differential expression of miRNAs between stretched and unstretched alveolar epithelial cells. We have further categorized these miRNAs by their number and types of predicted mRNA targets, and their functional annotations, providing a foundation for future investigations on the role of miRNAs in regulating the RAEC response to cyclic stretch. Finally, we have demonstrated a link between two miRNAs and the effect of their inhibition on stretch-induced epithelial permeability, providing one more mechanistic level of control for this pathogenic phenotype.

## Misc

Nadir Yehya and Adi Yerrapureddy designates joint first authorship

## Competing interests

The authors declare no competing interests.

## Authors’ contributions

NY contributed to study design, miRNA inhibitor transfection and permeability experiments, and was responsible for data analysis and interpretation after array normalization, as well as for writing the manuscript. AY was responsible for cell isolation from rat lungs, cell culture, RNA isolation, and RT-PCR validation, and contributed to study design and to writing the manuscript. JT was responsible for array normalization and bioinformatics. SSM was primarily responsible for study design and implementation, data interpretation, and for writing parts of the manuscript. All authors read and approved the final manuscript.

## Author's information

Nadir Yehya and Adi Yerrapureddy designates joint first authorship.
